# Crystal search – feasibility study of a real-time deep learning process for crystallization well images

**DOI:** 10.1107/S2053273323001948

**Published:** 2023-06-02

**Authors:** Yvonne Thielmann, Thorsten Luft, Norbert Zint, Juergen Koepke

**Affiliations:** a Molecular Membrane Biology, Max Planck Institute of Biophysics, Max-von-Laue-Strasse 3, Frankfurt am Main, 60438, Germany; b Systrade GmbH, Bockenheimer Landstrasse 47, Frankfurt am Main, 60325, Germany; University of Patras, Greece

**Keywords:** biocrystallization, high-throughput screening, deep learning, neural network, U-Net, AlexNet, VggNet, ResNet, SqueezeNet, BOINC

## Abstract

To avoid the time-consuming and often monotonous task of manual inspection of crystallization plates, a Python-based program to automatically detect crystals in crystallization wells employing deep learning techniques was developed. The program uses manually scored crystallization trials deposited in a database of an in-house crystallization robot as a training set. Since the success rate of such a system is able to catch up with manual inspection by trained persons, it will become an important tool for crystallographers working on biological samples. Four network architectures were compared and the SqueezeNet architecture performed best. In detecting crystals AlexNet accomplished a better result, but with a lower threshold the mean value for crystal detection was improved for SqueezeNet. Two assumptions were made about the imaging rate. With these two extremes it was found that an image processing rate of at least two times, but up to 58 times in the worst case, would be needed to reach the maximum imaging rate according to the deep learning network architecture employed for real-time classification. To avoid high workloads for the control computer of the CrystalMation system, the computing is distributed over several workstations, participating voluntarily, by the grid programming system from the Berkeley Open Infrastructure for Network Computing (BOINC). The outcome of the program is redistributed into the database as automatic real-time scores (ARTscore). These are immediately visible as colored frames around each crystallization well image of the inspection program. In addition, regions of droplets with the highest scoring probability found by the system are also available as images.

## Abstract 2

Four deep learning architectures were applied and SqueezeNet scored best. It was combined with the grid programming system BOINC to realize automatic real-time scoring of crystallization well images. Scores are written to a database and displayed to facilitate image inspection for users.

## 1. Introduction

X-ray crystallography is the traditional method to determine atomic structures of macromolecules and is still the dominant technique based on the number of PDB (Protein Data Bank) entries in 2020 (RCSB Protein Data Bank, 2021 ▸). The resolution revolution in cryogenic electron microscopy (cryo-EM) accelerated the determination of molecular structures (Kühlbrandt, 2014 ▸); however, single-particle cryo-EM is fast becoming a rival technique. Interestingly, purification conditions optimized for cryo-EM structure determination often show initial crystals in crystallization attempts (Stark & Chari, 2016 ▸; Chari *et al.*, 2015 ▸). However, despite methodological advances (Birch *et al.*, 2018 ▸), crystallization and the phasing problem remain the bottlenecks of macromolecular X-ray crystallography. To overcome the obstacle of crystallization itself, automatic high-throughput crystallization robots were introduced (Thielmann *et al.*, 2012 ▸) but the inspection of thousands of crystallization images still remains a burden.

Starting with edge detection in 1991 from robotically imaged crystallization trials (Ward *et al.*, 1988 ▸; Zuk & Ward, 1991 ▸), crystallization image analysis has been subject to research. Research projects have focused on edge-based features (Wilson, 2002 ▸; Bern *et al.*, 2004 ▸), texture analysis (Cumbaa & Jurisica, 2010 ▸; Ng *et al.*, 2014 ▸) and spectral methods (Walker *et al.*, 2007 ▸). This led to the classification of images into different classes based on local or global features. Additionally, different machine learning techniques have been used such as naive Bayes classifier (Wilson, 2002 ▸), support vector machines (Kawabata *et al.*, 2006 ▸) or neural networks (Walker *et al.*, 2007 ▸; Szegedy *et al.*, 2016 ▸). Training sets based on human classification have been published by Snell *et al.* (2008 ▸) and Rosa *et al.* (2018 ▸). The number of classes used and classification in these classes still seem to depend on personal preferences.

Attempts to classify crystallization experiments into as many as ten classes with three different categories of precipitate gave, according to Wilson (2006 ▸), worse results than the reduced number of seven classes. A total of 16 crystallographers classified 1207 images with an agreement for single crystals of 84.7% and 91.6% for empty drops and a weighted mean over all classes of 69.5% agreement. This indicates an agreement ratio between any two crystallographers of only about 70%. Snell *et al.* (2008 ▸) even found agreement between the middle and the end of their study differing by 7% and crystallographers tend to be biased when scoring their own experiments (Bruno *et al.*, 2018 ▸). Therefore, researchers have been looking for some time for algorithms to support manual evaluation or to make it completely redundant. The Machine Recognition of Crystallization Outcomes (MARCO, https://marco.ccr.buffalo.edu/) initiative claims to reach an accuracy exceeding 94% with their deep learning attempt based on the Inception-v3 architecture (Bruno *et al.*, 2018 ▸).

Already in 2010 the BOINC (Berkeley Open Infrastructure for Network Computing) system (Anderson, 2004 ▸) was used by the Help Conquer Cancer (HCC) project for automatic scoring of images from high-throughput protein crystallization trials (Cumbaa & Jurisica, 2010 ▸; Kotseruba *et al.*, 2012 ▸). Public resource or volunteer computing uses internet-connected computers whose owners voluntarily share unused capacities with scientists having a huge computational demand but low budget. Alternatively, grid or cloud computing share the same goal of better utilization of existing computing resources but have the advantage of being highly reliable as they are managed by IT professionals, while volunteer computers might get disconnected or shut down eventually.

Our goal was to use our existing crystallization image database, which represents a quite diverse test set, in two ways. The database encompasses crystallization drop images of soluble and membrane proteins from initial screening to crystals for high-resolution structure determination. These images were scored by users with different levels of experience, from graduate student to skilled researcher. This database was utilized to train and test different deep learning architectures. By application of the BOINC approach to our best results a pipeline was established to create an ARTscore (automatic real-time scores) process. The ARTscore is presented as a colored frame around the crystallization well image and droplet regions with highest scores are highlighted to facilitate the inspection by our users.

## 2. Methods

### 2.1. Training and testing set

For the training and validation sets we were able to make use of all 33 872 manually classified images available in our Rigaku CrystalMation database as at 24th October 2018. Images were scored according to the 13 classes shown in Table 1 ▸, with the exception of the class rim as described in Section 2.2. All images were further processed for use in deep learning attempts as described below. No further visual inspection of the images was performed to control the quality of the scoring by the users of our platform. In the case where only five classes were used, the images were summarized for score numbers 23, 22 and 21 to crystal, for 13, 12 and 11 to precipitate, for 34 to 31 to other, 1 and 2 to clear and rim as the fifth class.

Images are produced by the three Rigaku Automation Minstrel HT UV imagers of our fully automated CrystalMation crystallization platform and stored in an Oracle database (Thielmann *et al.*, 2012 ▸). Users access their images via *XtalTrak* (Rigaku Automation, Carlsbad, USA). Best focused images of the crystallization wells generated from four slices and one UV image are accumulated at a speed of about 16.25 s per well. With our standard procedure each crystallization plate is kept in the incubators for 3 months and imaged ten to 12 times during this period. If the imagers work without interruption through this schedule, the incubators could only be occupied with 207 plates. But if each of the six incubators is loaded with the maximum of 650 crystallization plates, our platform would have to be capable of imaging a well in just 5.19 s. To achieve this imaging rate, for example the number of slices has to be reduced. To keep up with this outcome of images, a program capable of classifying images in real time must be able to achieve this image processing rate. If the program needs a longer time *t* (s per well) for its calculations a parallelization of *t*/16.25 or up to *t*/5.19, respectively, would be necessary to keep pace. Therefore, the aim of this study was to set up a system capable of handling the necessary image processing rate.

### 2.2. Image preprocessing

Since the original images produced by the imagers have dimensions of 2452 × 2056 pixels and deep learning networks are usually capable of processing images with about 256^2^ pixels, the image size had to be reduced considerably. Initially the size could be shrunk by searching for the droplet inside the well, but this did not reduce the dimensions of the image far enough. Thus, in addition, the problem had to be sliced into smaller chops. This could either be done by shifting a smaller window in each direction in small steps over the larger image or by calculation of the smallest overlap necessary to cover the whole image by these smaller chops. Both attempts were used here.

To calculate the smallest overlap with *h_I_* and *w_I_*, the height and width of the image *I*, and *h_C_* and *w_C_*, the height and width of the chop *C*, the numbers of overlaps are the integer divisions *n* = *h_I_*/*h_C_* and *m* = *w_I_*/*w_C_* in the two dimensions. The distance *d* of the two overlaps of each chop become 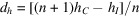 and 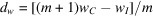 , respectively. For the *i* chops in height the chops start at 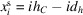 and end at 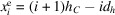 while the *j* chops in width start at 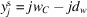 and end at 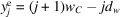 . This attempt was used to prepare chops of the droplets used for the training of the network.

To search for the droplets inside the well another deep learning approach was used. Segmentation of the input image is achieved with the U-Net architecture by coupling a convolution network with a deconvolution network (Ronneberger *et al.*, 2015 ▸; Zhixu, 2018 ▸). The contracting path is a typical convolutional network that consists of repeated application of convolutions, each followed by a rectified linear unit (ReLU) and a maximum pooling operation. During the contraction, the spatial information is reduced while feature information is increased. The expansive pathway combines the feature and spatial information through a sequence of up-convolutions and concatenations with high-resolution features from the contracting path. The graphic program *gimp* was employed to annotate a training set of 214 masks around the droplets found in these images. The output of the U-Net training is an hdf5 file (hierarchical data format version 5, https://www.hdfgroup.org/solutions/hdf5/), which will be used later in the classification process.

In a next step the images cropped to the size of the droplet are used to prepare smaller chops, useful for training with the four different network architectures. The droplet images are chopped according to the above smallest overlap algorithm and contours inside these chops are used for various tasks. Images classified by crystallographers as containing crystals are checked together with their UV mate for contours. Only when both contours were above a threshold were the images accepted as protein crystal images for training. Unfortunately, the number of UV pairs was much smaller than the scored images found before (Table 1 ▸). Also chops classified as precipitate (score Nos. 11–13) or other (score Nos. 31–34) were only accepted when their threshold was exceeded to avoid an overweighting of these classes.

We observed in first runs frequent misinterpretations of chops containing a part of the droplet rim as the class crystals harvestable (score No. 23). To avoid this misinterpretation in a first attempt, chops at the image border were given an individual threshold which could be higher than the one for chops of the image center. Unfortunately, this measure was not very successful. Much better was the attempt to select from images classified by the users of the CrystalMation system as clear drops. The chops from the image border which contained contours above a threshold were used to set up the new class called rim. It had the same score number 1 as the class clear. After the introduction of this new class rim for deep learning, the number of misinterpretations remained insignificant.

### 2.3. Network architectures

Four deep learning architectures were tested according to their performance for a real-time deep learning classification of the images extracted from our database.

The deep convolutional neural network (CNN) developed by Krizhevsky *et al.* (2012 ▸) of the University of Toronto for the ImageNet Large-Scale Visual Recognition Challenge (ILSVRC) (Russakovsky *et al.*, 2015 ▸) in 2010 is usually named after the first author, AlexNet. The network contains five convolutional and three fully connected layers with weights which finally feed into softmax. The depth of the AlexNet network means it is computationally expensive, but it is made feasible due to the utilization of GPUs during training. AlexNet was originally written in CUDA to run with GPU support.

An early attempt to generate deeper convolutional network architectures was the work of the Visual Geometry Group (Vgg) from the University of Oxford for ILSVCR-2014 (Simonyan & Zisserman, 2015 ▸). They started with 11 and increased their network successively to 13, 16 and 19 weight layers. With the number of layers increasing the number of filters learned increased as well, doubling with every maximum pooling applied. To train 16 or even 19 layers was very time consuming at that time and resulted in a huge model for only a poor gain. In this work we applied a VggNet of depth 16.

The depth of a network remained of central importance in many visual recognition tasks. Residual network (ResNet) architectures were constructed to allow for substantially deeper networks and these types of networks can gain higher accuracy from their increased depth (He *et al.*, 2015 ▸). To circumvent the degradation error which generates a higher error with the increased number of layers, the authors have introduced shortcut connections skipping one or more layers and performing identity mapping added to the output of the shortcut stacked layers. We used here a ResNet with 50 weight layers (*i.e.* convolutional or fully connected layers) prior to the final softmax classifier.

The SqueezeNet architecture was developed to reduce the model size while keeping the possible accuracy that can be reached currently with modern CNNs (Iandola *et al.*, 2017 ▸). The aim of this architecture was to reach an accuracy comparable with that of AlexNet with the ILSVRC-2012 ImageNet data set (Deng *et al.*, 2009 ▸). The model size reduction is achieved by drastically reducing the number of parameters from 60 million for AlexNet to 1.2 million, and the subsequent usage of deep compression (Han *et al.*, 2016 ▸) reduced the number of parameters even further to 421 098. In Table 2 ▸ the model size, the number of weight layers and the computation times of the investigated networks are listed. Compared with AlexNet we got a model size reduction of nearly 77 times with SqueezeNet for our data.

The Python implementations of Rosebrook (2017 ▸) were used to install the four networks on our workstations. They utilize multiple GPU support by employing the MXNet library (Chen *et al.*, 2015 ▸). Our data were processed with the *MXNet im2rec* tool to create efficiently packed record files which reduced the file size of the training set by a factor of 3.4 compared with a respective hdf5 file. All our training attempts were carried out on workstations hosting four NVIDIA TITAN Xp GPUs. Learning was started with a learning rate of 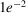 , subsequently lowered stepwise to 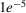 and stopped after stagnation with the number of epochs listed in Table 2 ▸. The accuracy reached and loss function for training and validation sets are listed in additional columns.

To understand the deep learning performance better, we compared the two best architectures with each other and also used the database of the MARCO initiative for this (MARCO, 2018 ▸).

### 2.4. BOINC installation

In order to ensure the unrestricted operation of a program on the computers of volunteers, executables for all used operating systems must be provided on the BOINC server. Python is an interpreted language; therefore the script needs to be frozen in the Python version used, with all libraries and modules used by a certain tool becoming independent from the Python installation. Only the freezing tools *cx_Freeze* and *pyInstaller* work on all three operating systems Linux, Windows and OsX, and only *pyInstaller* is able to freeze the code into a single executable file (The Hitchhiker’s Guide to Python: Freezing Your Code, https://docs.python-guide.org/shipping/freezing/). The Python libraries *skimage* and *MXNet* prevent the freezing of our code with these tools. Therefore, we are currently restricted to BOINC participants that have *miniconda* installed (https://docs.conda.io/en/latest/miniconda.html), to keep the demands (download and disk space of 5 GBytes with all libraries) on the clients low.

The data preparation for processing in BOINC is realized with scripts and based on a client/server architecture. The BOINC server comes by default with the core components scheduler, feeder, transitioner, validator, assimilator and file deleter (Fig. S1 in the supporting information). Its installation in a docker container combines a single application (BOINC) with all dependencies like libraries, utilities and static data in one image file (Docker, 2019 ▸; BOINC Project Cookbook, 2019 ▸). Therefore, containers can be compared with a lightweight virtualization. With the docker container, the complete BOINC server, including web application and database, is independently stored and transportable by the operating system. This means that all running core components within the docker container can be reset or reinstalled at any time. The core components start automatically after the start of the container and are ready for operation. The transitioner provides the archive and application in the download directory for the clients to upload. The scheduler distributes the work packages to the clients and the validator examines the result after processing. The assimilator (customized script) stores the results in the results directory before the file deleter deletes them from BOINC.

## 3. Results

### 3.1. Automatic scoring

**Figure 1:**
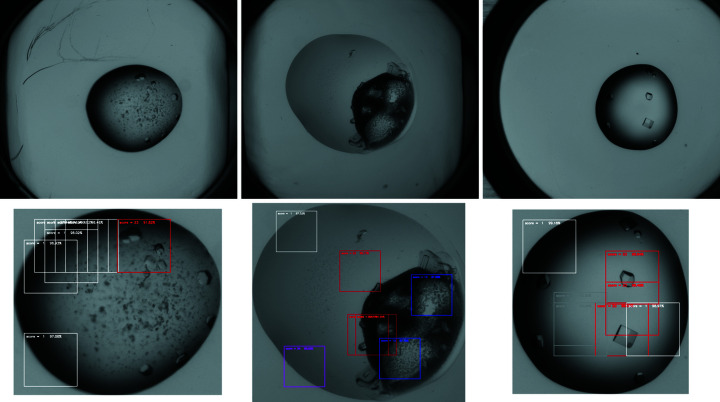
Top row: original crystallization well images extracted from the Oracle database. Bottom row: processed images reduced to the droplet size with ARTscores highlighting the chops with highest probability for a certain class (red, crystal; blue, precipitate; white, rim; gray, clear; magenta, null experiment).

For the evaluation of the crystallization plates, a Python program was written that utilizes first the U-Net model to locate the droplet inside a well and second the MXNet model to classify chops of the droplet image. The chop was shifted in this program by steps of *s* pixels across the image and evaluated according to the MXNet model at each of these positions. Steps with *s* = 50 pixels gave the best results in our hands. In a next program step the outcome of these chops was scored according to a scheme ranking from high to low in the following order: crystal classes highest, precipitate and other next, and clear with the lowest priority. If a crystal class is found, its score is accounted for the whole image and the same happens to the other classes in descending order. All scores in this procedure have further to exceed a threshold *c* which was set to 85% accuracy. Colored frames around the chops with the *n* highest scores are drawn into an output image (Fig. 1 ▸, Fig. S2).

To validate the classifications the four networks achieved, 16 crystallization plates were completely scored in one inspection by volunteers and their scoring into the classes crystal, precipitate, other and clear was compared with the outcome of the four network classifications. The hits listed in the supporting information files CrystalSearchSuppl1.xslx (all network architectures) and CrystalSearchSuppl.2.xslx (comparison of AlexNet only) were then used to calculate success rates, which are listed in a column next to the hits. The total weighted means were calculated from the individual weighted means of each class, weighted according to their frequency, and finally listed in Table 3 ▸. With a weighted mean of 77.1% over all classes for the 16 test crystallization plates, SqueezeNet gave in our hands the best results. But with success rates for crystal detection of up to 90% for three of the crystallization plates and 76.9% in the mean for the 2400 images, AlexNet scored better. Only when we lowered the above threshold *c* to 80% accuracy for SqueezeNet could the mean value for crystal detection be improved from 69.9% to the second best value of 74.3%, while the overall weighted mean changed only insignificantly to 77.0%.

If AlexNet was trained in our installation with only five possible classes, the performance on the class crystal decreased to only 64.1% and also a decreased weighted mean for all classes was the result (68.3% compared with 74.2% for 13 classes). If AlexNet was trained with the MARCO database the success rate on crystal detection increased to 81.4% but the classes precipitate and other only matched in 37.1% and 7.4% of cases, and the overall weighted mean decreased to 69.4%.

### 3.2. Image processing rate

Based on an 8 CPU workstation, it was possible to calculate the time necessary to evaluate a well of a crystallization plate in the respective network as shown in Table S1. According to the considerations made earlier, the parallelization shown in the last two rows of the table would be necessary to keep pace with the appearance of new crystallization plate images, either in the standard setup with 16.25 s per well (penultimate row) or with the highest possible imaging rate of 5.19 s per well (last row).

To reach this grade of image processing rate BOINC was employed to guarantee a real-time processing of the images with the favored network architectures AlexNet or Squeeze­Net. The participation of the computers available in our internal IT network was sufficient. All these machines had the same Python environment installed. A much higher parallelization would only be required if we wished to process all 15 666 284 (17th October 2019) crystallization images currently available in our CrystalMation database, *e.g.* a parallelization of 183 or 216 to finish the processing of all database entries based on AlexNet or SqueezeNet, respectively, within a month.

To run the evaluation program under the regime of BOINC, additional scripts were written. The script *create_work4cf.py* queries search patterns for image types and generates an SQL command from them, which extracts the corresponding information from the Oracle database. In the second part of the script the image names are compared with files in the file system and the images are packed into ZIP files (archives) and stored in the file system of the BOINC server. Another script *finalizer-2dir.py*, outside the BOINC container, checks the existing results and the score in the Oracle database. The evaluated score is stored in a CSV file for later processing. This step was introduced to avoid annoying users with false positives.

The scripts *set_autoscore-dir.py* and *finalizer-2dir.py* are started as a service and work fully automatically. Images generated by the evaluation program showing frames with probabilities for highest scoring areas of the crystallization droplet (Fig. 1 ▸) can not yet be written into the database by the second script. These images would be helpful to analyze cases where *e.g.* the evaluation fails.

The script *set_autoscore-dir.py* is the last step in the process chain. It takes the CSV file created by the finalizer and imports the results into the score table of the Oracle database of *XtalTrak*. Here the ARTscore is visible for users to assist in image classification.

## 4. Discussion

We were successful in creating a fully automatic programmed system able to process the images created by our CrystalMation system in real time by employing only in-house workstations.

Since the image scoring of our training set was generated from all the users of our CrystalMation system for their own purposes (about 40 researchers, from graduate to experienced crystallographer) the scoring might be quite heterogeneous. It is probably not as unambiguous as the scoring for the MARCO database (MARCO, 2018 ▸). Therefore it was interesting to compare the learning accuracy of the four network architectures and combine the best of our installation with the database of MARCO data.

AlexNet and SqueezeNet performed best with our database with a success for crystals and a weighted mean of 76.9% and 74.2% for AlexNet and 74.3% and 77.0% for SqueezeNet, respectively. If the training of AlexNet was performed with five instead of 13 classes the success rates for the class crystal and also the weighted mean on all classes decreased. Most interestingly, the accuracy on the class crystal and on the class clear dropped by about 12 percentage points (see CrystalSearchSuppl2.xlsx in the supporting information). When we compared AlexNet trained with our database and the MARCO database, the success rate on the class crystal increased clearly. But the overall weighted mean decreased considerably as the classes precipitate and other were only successfully identified in 37.1% and 7.4% of cases, respectively. The training on the MARCO database did not match these classes of images of our CrystalMation system. There can be several reasons for this, *e.g.* different drop appearance (vapor diffusion versus under oil crystallization), drop shape (different for membrane and soluble proteins), different imaging systems, also different granular or gel-like appearances of precipitates and phase separations and different considerations on how to use the class other.

The evaluation of UV images for the class crystal might have prevented an overfitting of the class crystal in the current work, but it has to be critically rethought. For salt crystals it might work very well to exclude these during image evaluation. But for crystals with chromophores it might be a disadvantage as their UV mate might not show a crystal as well, but this result should not be excluded. It could be evaluated manually by the inspection of colored images, which are regularly taken for colored samples.

Manually created scores may not be overwritten; therefore, the automatic evaluations are dated to the time of the respective inspection. This ensures that they are younger than any possible manual rating and can therefore not overwrite them. However, manual evaluations of older inspections are overwritten by the automatic scoring. This is a serious problem. Here, it would be desirable to keep both evaluations with the image for later considerations. Drop regions with highest scores should be displayed as additional information.

To process the images of the whole database a much higher parallelization is necessary. This is the reason why we need to succeed in freezing the evaluation program into a single executable. When we can provide executables for all common operating systems, the evaluation program can be distributed to enough clients. This is necessary to process the image files in a reasonable timescale. Unfortunately, our efforts have so far ended up in extremely large executable files of up to 5 Gbytes size, if all necessary libraries are included. This is too large to be handled by the BOINC clients. In future attempts this problem can be overcome by translating the Python code with a tool like *Nuitka* (*Nuitka User Manual*, https://nuitka.net/doc/user-manual.html) into a C^++^ program which is compiled in a next step into a standalone executable. In addition, the use of C^++^ will lead to a speed gain of currently 258% (Hayen, 2023 ▸).

By working on our original goal of determining the level of parallelization required, we made an unexpected discovery: our program creates by accident the ideal conditions to gain the ground-truth bounding boxes for a future project with faster regions with CNN features (R-CNN). Reprogramming our evaluation program at the scoring process will enable us to output the respective best 256^2^ chops as bounding boxes and to distribute them to the appropriate class folders instead of having the tedious work of hand labeling. Further improvement of the Python code itself could be achieved through the use of R-CNN (Girshick *et al.*, 2014 ▸). The use of R-CNN techniques could lead to a dramatically higher object detection performance compared with the simple sliding windows algorithm used here.

## 5. Conclusion

The deep learning architecture SqueezeNet was used to establish an automatic scoring algorithm to simplify the scoring of our crystallization images in the database for our users. More than 74% of the crystals were detected by the best performing algorithm SqueezeNet with an overall accuracy of 77.0% on all classes. The output of SqueezeNet is the ARTscore. It is displayed as a colored frame around the crystallization image and can be used to facilitate the screening for crystals. It can also be used to score all unscored crystallization conditions left in the database. Once these are tagged with the ARTscore for the class crystal they are available for database screening of successful crystallization conditions. These could be used to design smarter crystallization conditions based on different protein superfamilies (for membrane proteins) or the outcome could even be used to predict new crystallization conditions (for soluble proteins).

The current algorithm was trained on vapor diffusion drops. If microbatch or *in meso* crystallization drops were included in a similar number compared with vapor diffusion drops in the training set, these images could also be applicable to the algorithm. Microbatch or *in meso* crystallization might need adaptations for the special appearance of the drops in each case, as was done for the class rim for the identification of the drop edge in this study. It might be very useful to focus more strongly on the use of UV crystallization images as artifacts from different plate shapes and surface borders are less pronounced in these images.

## Supplementary Material

CrystalSearchSuppl1.xlsx. DOI: 10.1107/S2053273323001948/ik5007sup1.xlsx

CrystalSearchSuppl2.xlsx. DOI: 10.1107/S2053273323001948/ik5007sup2.xlsx

Supporting figures and table. DOI: 10.1107/S2053273323001948/ik5007sup3.pdf

## Figures and Tables

**Table 1:** Overview of all images used in deep learning distributed into the different classes of the CrystalMation system Classes are ranked from high to low in priority. The class rim was created for image preprocessing only. Other features in the class ‘clear with other features’ might be a fiber or a scratch, for example.

Class	Score No.	Images	UV pairs	Chops
Crystals harvestable	23	3428	2527	21164
Crystals bad form	22	2123	1091	7733
Crystals overnucleated	21	2873	1409	14381
Precipitate	13	10994		56534
Precipitate bad	12	1142		13593
Precipitate good	11	1459		9569
Null experiment	34	515		2608
Other	33	788		7974
Spherolites	32	189		813
Phase separation	31	1946		7326
Clear	1	7613		159687
Clear with other features	2	802		4603
Rim	1			18666
Total		33872		324651

**Table 2:** Training and validation accuracy of four network architectures for the 13 classes listed in Table 1 ▸ The table also includes the training and validation accuracy for our attempts to use only five classes for AlexNet as well as the MARCO data. The loss function (Loss) is used to optimize a deep learning algorithm, thus the smaller the loss the better the prediction fits the data. The accuracy (Acc.) is a measure of how many predictions were correct compared with the total number of predictions. Top5 is the accuracy for only the 5 best predicted labels of the model.

	Model size	Weight	No. of	Total time	Time per	Training (%)			Validation (%)		
Network architecture	(MB)	layers	epochs	(h:min:s)	epoch (min)	Acc.	Top5	Loss	Acc.	Top5	Loss
AlexNet (13)	223	8	100	04:14:20	2.54	83.7	98.9	0.48	78.2	97.4	0.73
AlexNet (5)						83.5		0.45	82.3		0.51
AlexNet (MARCO)						90.6		0.26	88.5		0.32
VggNet	513	16	70	15:08:17	12.98	78.0	97.1	0.68	76.5	96.5	0.77
ResNet	90	50	55	05:32:02	6.04	85.9	99.2	0.41	79.5	97.7	0.66
SqueezeNet	2.9	18	200	08:26:48	2.53	81.7	98.3	0.54	79.4	97.6	0.64

**Table 3:** Mean success rates (%) of the four employed network architectures for the classes crystal, precipitate, other and clear The overall weighted means of the four network architectures are listed in the rightmost column. AlexNet was trained with 13 and five classes for our own data and in addition the MARCO data.

Architecture	Crystal	Precipitate	Other	Clear	Weighted mean
AlexNet (13 classes)	76.88	69.81	55.48	85.10	74.16
AlexNet (5 classes)	64.09	71.33	51.04	73.76	68.33
AlexNet (MARCO)	81.42	37.11	7.39	67.56	69.42
VggNet	60.05	73.53	54.24	71.18	68.07
ResNet	72.44	66.31	62.03	77.26	70.05
SqueezeNet (*c* = 85)	69.86	78.55	67.14	89.95	77.09
SqueezeNet (*c* = 80)	74.31	76.46	66.77	87.96	77.00

## References

[bb19] Anderson, D. P. (2004). *BOINC: A System for Public-Resource Computing and Storage*. Fifth IEEE/ACM International Workshop on Grid Computing, Pittsburgh, PA, USA, pp. 4–10. https://doi.org/10.1109/GRID.2004.14.

[bb20] Bern, M., Goldberg, D., Stevens, R. C. & Kuhn, P. (2004). *J. Appl. Cryst.* **37**, 279–287.

[bb21] Birch, J., Axford, D., Foadi, J., Meyer, A., Eckhardt, A., Thielmann, Y. & Moraes, I. (2018). *Methods*, **147**, 150–162. 10.1016/j.ymeth.2018.05.014 29778646

[bb14] BOINC (2019). BOINC Project Cookbook, https://github.com/marius311/boinc-server-docker/blob/master/docs/cookbook.md.

[bb22] Bruno, A. E., Charbonneau, P., Newman, J., Snell, E. H., So, D. R., Vanhoucke, V., Watkins, C. J., Williams, S. & Wilson, J. (2018). *PLoS One*, **13**, e0198883. 10.1371/journal.pone.0198883 PMC6010233 29924841

[bb23] Chari, A., Haselbach, D., Kirves, J.-M., Ohmer, J., Paknia, E., Fischer, N., Ganichkin, O., Möller, V., Frye, J. J., Petzold, G., Jarvis, M., Tietzel, M., Grimm, C., Peters, J.-M., Schulman, B. A., Tittmann, K., Markl, J., Fischer, U. & Stark, H. (2015). *Nat. Methods*, **12**, 859–865. 10.1038/nmeth.3493 PMC5136620 26237227

[bb8] Chen, T., Li, M., Li, Y., Lin, M., Wang, N., Wang, M., Xiao, T., Xu, B., Zhang, C. & Zhang, Z. (2015). arXiv:1512.01274.

[bb25] Cumbaa, C. A. & Jurisica, I. (2010). *J. Struct. Funct. Genomics*, **11**, 61–69. 10.1007/s10969-009-9076-9 PMC2857471 20072819

[bb26] Deng, J., Dong, W., Socher, R., Li, L.-J., Li, K. & Fei-Fei, L. (2009). *IEEE Computer Society Conference on Computer Vision and Pattern Recognition*, pp. 248–255.

[bb13] Docker (2019). Docker Documentation, https://docs.docker.com.

[bb17] Girshick, R., Donahue, J., Darrell, T. & Malik, J. (2014). arXiv:1311.2524.

[bb7] Han, S., Mao, H. & Dally, W. J. (2016). arXiv:1510.00149v5.

[bb16] Hayen, K. (2023) *Nuitka*. https://nuitka.net.

[bb5] He, K., Zhang, X., Ren, S. & Sun, J. (2015). arXiv:1512.03385.

[bb6] Iandola, F. N., Han, S., Moskewicz, M. W., Ashraf, K., Dally, W. J. & Kreutzer, K. (2017). arXiv:1602.07360.

[bb27] Kawabata, K., Takahashi, M., Saitoh, K., Asama, H., Mishima, T., Sugahara, M. & Miyano, M. (2006). *Acta Cryst.* D**62**, 239–245. 10.1107/S0907444905041077 16510970

[bb28] Kotseruba, Y., Cumbaa, C. A. & Jurisica, I. (2012). *J. Phys. Conf. Ser.* **341**, 012027.

[bb29] Krizhevsky, A., Sutskever, I. & Hinton, G. E. (2012). *Advances in Neural Information Processing Systems* 25, edited by F. Pereira, C. J. C. Burges, L. Bottou & K. Weinberger, pp. 1097–1105, https://papers.nips.cc/paper/2012/hash/c399862d3b9d6b76c8436e924a68c45b-Abstract.html

[bb30] Kühlbrandt, W. (2014). *Science*, **343**, 1443–1444. 10.1126/science.1251652 24675944

[bb10] MARCO (2018). MARCO database, https://ubir.buffalo.edu/xmlui/handle/10477/77793.

[bb31] Ng, J. T., Dekker, C., Kroemer, M., Osborne, M. & von Delft, F. (2014). *Acta Cryst.* D**70**, 2702–2718. 10.1107/S1399004714017581 PMC4188010 25286854

[bb1] RCSB Protein Data Bank (2021). *Statistics on all Released Structures*. https://www.rcsb.org/stats/all-released-structures.

[bb32] Ronneberger, O., Fischer, P. & Brox, T. (2015). In *Medical Image Computing and Computer-Assisted Intervention*, edited by N. Navab, J. Hornegger, W. Wells & A. Frangi. MICCAI 2015. Lecture Notes in Computer Science, Vol. 9351. Cham: Springer.

[bb33] Rosa, N., Ristic, M., Marshall, B. & Newman, J. (2018). *Acta Cryst.* F**74**, 410–418. 10.1107/S2053230X18008038 PMC6038447 29969104

[bb34] Rosebrook, A. (2017). *Deep Learning for Computer Vision with Python: Image Net Bundle*, Vol. 3. PyImageSearch.

[bb35] Russakovsky, O., Deng, J., Su, H., Krause, J., Satheesh, S., Ma, S., Huang, Z., Karpathy, A., Khosla, A., Bernstein, M., Berg, A. C. & Fei-Fei, L. (2015). *Int. J. Comput. Vis.* **115**, 211–252.

[bb4] Simonyan, K. & Zisserman, A. (2015). arXiv:1409.1556.

[bb36] Snell, E. H., Luft, J. R., Potter, S. A., Lauricella, A. M., Gulde, S. M., Malkowski, M. G., Koszelak-Rosenblum, M., Said, M. I., Smith, J. L., Veatch, C. K., Collins, R. J., Franks, G., Thayer, M., Cumbaa, C., Jurisica, I. & DeTitta, G. T. (2008). *Acta Cryst.* D**64**, 1123–1130. 10.1107/S0907444908028047 PMC2631114 19020350

[bb37] Stark, H. & Chari, A. (2016). *Microscopy (Tokyo)*, **65**, 23–34. 10.1093/jmicro/dfv367 26671943

[bb38] Szegedy, C., Vanhoucke, V., Ioffe, S., Shlens, J. & Z. Wojna, Z. (2016). *Proceedings of the IEEE Conference on Computer Vision and Pattern Recognition*, pp. 2818–2826.

[bb39] Thielmann, Y., Koepke, J. & Michel, H. (2012). *J. Struct. Funct. Genomics*, **13**, 63–69. 10.1007/s10969-011-9118-y 22101889

[bb40] Walker, C. G., Foadi, J. & Wilson, J. (2007). *J. Appl. Cryst.* **40**, 418–426.

[bb41] Ward, K. B., Perozzo, M. A. & Zuk, W. M. (1988). *J. Cryst. Growth*, **90**, 325–339.

[bb43] Wilson, J. (2002). *Acta Cryst.* D**58**, 1907–1914. 10.1107/s0907444902016633 12393921

[bb42] Wilson, J. (2006). *Advances in Data Mining, Applications in Medicine, Web Mining, Marketing, Image, Signal Mining*, edited by P. Perner, pp. 459–473. Berlin, Heidelberg: Springer.

[bb2] Zhixu, H. (2018). *Implementation of Deep Learning Framework – Unet, Using Keras*. https://github.com/zhixuhao/unet.

[bb24] Zuk, W. M. & Ward, K. B. (1991). *J. Cryst. Growth*, **110**, 148–155.

