# Supplementary material for: Crystal search – feasibility study of a real-time deep learning process for crystallization well images
Source: Acta Crystallogr A Found Adv. 2023 Jun 2;79(Pt 4):331–8. doi: 10.1107/S2053273323001948 (PMC10317135; doi:10.1107/S2053273323001948)
Supplement: Supplementary file 3 [file a-79-00331-sup3.pdf]

Acta Cryst A
FOUNDATIONS
ADVANCES

Volume 79 (2023)

Supporting information for article:

Crystal search – feasibility study of a real-time deep learning process of crystallization well images

Yvonne Thielmann, Thorsten Luft, Norbert Zint and Juergen Koepke

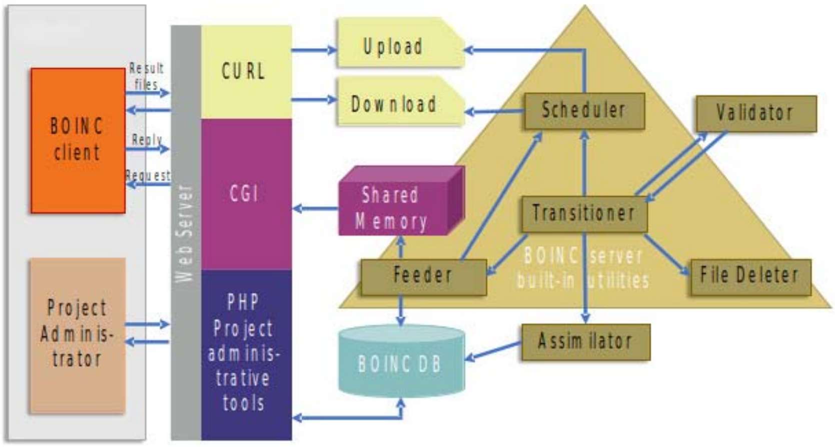

Figure S1 The basic structure of a BOINC project is based on a client/server architecture. Shown on the left side are the BOINC client and manager, applications installed on computers of voluntary project participants. And on the right hand side the BOINC server with a SQL database and the BOINC demons scheduler, feeder, transitioner, validator, assimilator, and file deleter (optional) are shown. The communication of the left side applications with the server is performed via an APACHE webserver.

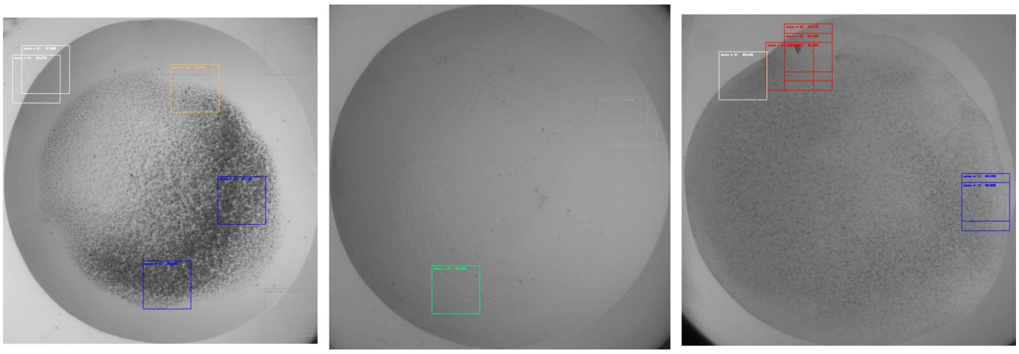

Figure S2 Processed images reduced to droplet size with ARTscores highlighting the chops with highest probability for a certain class (red crystal, orange crystals overnucleated, blue precipitate, white rim, grey clear, green phase separation).

Table S1 Calculation times of the evaluation program of four network architectures for 16 test crystallization plates.

**crystal. plate**		**AlexNet**		**VggNet**		**ResNet**		**SqueezeNet**	
	no.	total time	per img. [s]	total time	per img. [s]	total time	per img. [s]	total time	per img.[s]
**MP001152**	96	00:42:47	26.74	07:04:29	265.30	02:31:50	94.90	00:51:16	32.04
**MP001161**	96	00:27:14	17.02	04:07:48	154.88	01:30:29	56.55	00:32:17	20.18
**MP001163**	96	00:23:33	14.72	03:19:32	124.71	01:13:38	46.02	00:25:58	16.23
**MP001701**	96	00:20:04	12.54	02:57:50	111.15	01:04:08	40.08	00:23:30	14.69
**MP001925**	288	03:38:23	45.50	37:21:23	466.95	13:04:11	163.37	04:21:44	54.53
**MP002047**	288	03:46:21	47.16	38:48:38	485.13	13:42:43	171.40	04:31:55	56.65
**MP002148**	96	01:33:32	58.46	15:58:53	599.30	05:21:57	201.22	01:47:55	67.45
**MP002206**	288	03:46:02	47.09	38:31:59	481.66	13:35:20	169.86	04:30:03	56.26
**MP002267**	96	00:41:28	25.92	06:31:48	244.88	02:17:37	86.01	00:46:15	28.91
**MP002269**	96	00:40:32	25.33	06:21:53	238.68	02:15:34	84.73	00:45:48	28.63
**MP002270**	96	00:45:00	28.13	07:32:33	282.84	02:37:23	98.36	00:54:32	34.08
**MP002369**	96	00:23:35	14.74	03:21:12	125.75	01:12:55	45.57	00:25:49	16.14
**MP002368**	96	00:39:27	24.66	06:08:27	230.28	02:11:15	82.03	00:44:46	27.98
**MP002370**	96	00:37:08	23.21	05:58:02	223.77	02:08:55	80.57	00:43:15	27.03
**MP002412**	288	01:17:04	16.06	11:23:55	142.48	04:09:24	51.96	01:26:50	18.09
**MP002413**	288	01:04:21	13.41	09:47:05	122.31	03:33:28	44.47	01:15:55	15.82
**wgt. mean**			**29.96**		**296.04**		**104.59**		**35.28**
**standard parallelization**			2		19		7		3
**highest parallelization**			6		58		21		7
